# Determination of optimal parameters of MAFFT program based on BAliBASE3.0 database

**DOI:** 10.1186/s40064-016-2526-5

**Published:** 2016-06-16

**Authors:** HaiXia Long, ManZhi Li, HaiYan Fu

**Affiliations:** School of Information Science Technology, Hainan Normal University, Haikou, 571158 HaiNan China; School of Mathematics and Statistics, Hainan Normal University, Haikou, 571158 HaiNan China

**Keywords:** Multiple sequence alignment, Gap open penalties, Gap extension penalties, Substitution matrix, MAFFT program, Default parameters

## Abstract

**Background:**

Multiple sequence alignment (MSA) is one of the most important research contents in bioinformatics. A number of MSA programs have emerged. The accuracy of MSA programs highly depends on the parameters setting, mainly including gap open penalties (GOP), gap extension penalties (GEP) and substitution matrix (SM). This research tries to obtain the optimal GOP, GEP and SM rather than MAFFT default parameters.

**Results:**

The paper discusses the MAFFT program benchmarked on BAliBASE3.0 database, and the optimal parameters of MAFFT program are obtained, which are better than the default parameters of CLUSTALW and MAFFT program.

**Conclusions:**

The optimal parameters can improve the results of multiple sequence alignment, which is feasible and efficient.

## Background

Multiple sequence alignment (MSA), one of the most basic bioinformatics tool, has wide applications in sequence analysis, gene recognition, protein structure prediction, and phylogenetic tree reconstruction, etc. MSA computation is a NP-complete problem (Lathrop 1995), whose time and space complexity have sharp increase while the length and the number of sequences are increasing.

At present, many scholars have developed open source online alignment tools, such as CLUSTALW, T-COFFEE, MAFFT, (Thompson et al. 1994; Notredame et al. 2000; Katoh et al. 2002; Katoh and Toh 2008) and so on. Using these tools, the results of MSA can be quickly obtained, so the tools are mainly used in MSA. MSA programs have two kinds of parameters: substitution matrix (SM) and gap penalty. Gap penalties include gap open penalties (GOP) and gap extension penalties (GEP). Many scholars have discussed the parameters setting. Thompson et al. (1994) propose that SM are varied at different alignment stages according to the divergence of the sequences to be aligned. Residue-specific gap penalties and locally reduced gap penalties in hydrophilic regions encourage new gaps in potential loop regions rather than regular secondary structure. Reese and Pearson (2002) provides an empirical basis for selection of gap penalties and demonstrates how optimal gap penalties behave as a function of the target evolutionary distance of the substitution matrix. Madhusudhan et al. (2006) suggests the variable penalty formula according to the structure of sequence based on dynamic programming. But these formulae are not widely used. Gondro and Kinghorn (2007) think that the gap penalty parameters were determined by the experience.

How to determine that the optimum parameters have no theoretical framework at present. Different parameter combinations could result in different MSA. The majority of users use default parameters when applying these alignment tools, but the results could not be the best. In addition, there is no effective method to determine the optimal parameter directly, so it is difficult to get the local optimal solution through online tools. Pais et al. (2014) summarize the efficiency of MSA methods and tools, such as CLUSTALW, CLUSTAL OMEGA, DIALIGN-TX, MAFFT, MUSCLE, POA, PROBALIGN, PROBCONS and T-Coffee. They obtain the following conclusion: T-Coffee and MAFFT are more efficiency to MSA (Pais et al. 2014). Nuin et al. (2006) compared nine commonly used MSA programs: CLUSTAL W, Dialign2.2, T-Coffee, POA, muscle, MAFFT, PROBCONS, DIALIGN-T and KALIGN, and obtained the following conclusions: among the nine programs tested, the iterative approach available in MAFFT (L-INS-i) and PROBCONS were consistently the most accurate, with MAFFT being the faster of the two. The above analyses reveal that MAFFT is the best choice for protein sequence alignment based on its overall alignment quality and processing speed. Ahola et al. (2006) introduce a statistical score that assesses the quality of a given multiple sequence alignment, and compare the AQ (alignment quality) scores of the seven alignment methods using the BAliBASE as a benchmarking database. According to these results, the MAFFT strategy L-INS-i outperforms the other methods. These conclusions are described in Web page (MAFFT Version 6). The speed and accuracy of MSA are most important evaluation criteria. With development of CPU and GPU technology, computer hardware can improve the MSA speed, so improving accuracy of MSA is the main factor influencing the MSA. The paper tries to obtain the optimal parameters combining GOP, GEP and SM based on MAFFT program.

The accuracy of MSA is usually assessed by scores. A number of score functions exist for alignment optimization, e.g. weighted sum-of-pairs, maximum likelihood, minimum entropy, star, and consensus (Gotoh 1999). The most popular score function is the weighted sum-of-pairs score (WSP). The best known standard measures for the evaluation of multiple sequence alignments are sum-of-pair score (SPS) and column score (CS) defined in (Thompson et al. 1999). Ahola et al. (2006) propose that statistical score assesses the quality of a given multiple sequence alignment. In the Ref. (Francisco et al. 2015), a set of novel regression approaches are proposed for the MSA evaluation by comparing several supervised learning and mathematical methodologies.

## Methods

### MAFFT program

MAFFT is a high speed multiple sequence alignment program for unix-like operating systems. The software is named after the acronym multiple alignment using fast Fourier transform after the major computational technique used by the method (Katoh et al. 2002). Due to the increasing necessity for MSA of distant homologs, Katoh et al. (2005) sought to improve the accuracy of MAFFT in 2005, and released Version 5. In 2008 and 2013, Version 6 (Katoh and Toh 2008) and Version 7 (Kazutaka and Standley 2013) were released.

MAFFT (MAFFT-7.220-WIN64 version) offers various multiple alignment strategies. They are classified into three types, (a) the progressive method, (b) the iterative refinement method with the WSP score, and (c) the iterative refinement method using both the WSP and consistency scores. In general, there is a tradeoff between speed and accuracy. The order of speed is a > b > c, whereas the order of accuracy is a < b < c. The following are the detailed procedures for the major options of MAFFT illustrated in Table 1.

**Table 1:** MAFFT algorithms and parameters (substitution matrix is denoted by bl)

Method types	Algorithms	Parameters	Explain
Progressive methods	FFT-NS-1	gop 1.53 gep 0.123 bl 62 retree 1–maxiterate 0	Approximately two times faster than the default
	FFT-NS-2	gop 1.53 gep 0.123 bl 62 retree 2–maxiterate 0	The accuracy of the FFT-NS-2 is slightly higher than that of the FFT-NS-1
Iterative refinement method	FFT-NS-i	gop 1.53 gep 0.123 bl 62 retree 2–maxiterate 1000	Fastest in this category. Uses WSP score only
	NW-NS-i	gop 1.53 gep 0.123 bl 62 retree 2–maxiterate 0	Distance is by the 6mer method
Iterative refinement methods using WSP and consistency scores	L-INS-i	gop 1.53 gep 0.123 bl 62 retree 2–maxiterate 1000–localpair	Uses WSP score and consistency score from local alignments
	E-INS-i	gop 1.53 gep 0.123 bl 62 retree 2–maxiterate 1000–genafpair	Uses WSP score and consistency score from local alignments with a generalized affine gap cost
	G-INS-i	gop 1.53 gep 0.123 bl 62 retree 2–maxiterate 1000–globalpair	Uses WSP score and consistency score from global alignments

References prove that MAFFT-L-INS-i and E-INS-i show the highest accuracy scores in currently available sequence alignment programs. However, the difference among MAFFT-L-INS-i, E-INS-i, TCoffee and ProbCons is quite small and not statistically significant in most cases (Ahola et al. 2006; MAFFT Version 6; Gotoh 1999). From Table 1, we can find that GOP is 1.53, GEP is 0.123 and substitution matrix is Blosum62 in MAFFT-L-INS-i and E-INS-I algorithm. So, our study tries to obtain the optimal GOP, GEP and substitution matrix rather than MAFFT default parameters.

### Sum-of-pairs score (SPS)

To assess the performance of the parameters in this study, we use the SPS scores to estimate the quality of an alignment.

The sum-of-pairs score (SPS) function used in (Thompson et al. 1999). Suppose there is a test alignment of N sequences consisting of M columns, and designate the *i*th column in the alignment by ${\text{A}}_{{{\text{i}}1}} ,{\text{A}}_{{{\text{i}}2}} , \ldots ,{\text{A}}_{\text{iN}}$. For each pair of residues ${\text{A}}_{\text{ij}}$ and ${\text{A}}_{\text{ik}} ,$ we define ${\text{p}}_{\text{ijk}}$ such that ${\text{p}}_{\text{ijk}} = 1$ if residues ${\text{A}}_{\text{ij}}$ and ${\text{A}}_{\text{ik}}$ are aligned with each other in the reference alignment, otherwise ${\text{p}}_{\text{ijk}} = 0$. The score ${\text{S}}_{\text{i}}$ for the *i*th column is defined as

1 $${\text{S}}_{\text{i}} = \mathop \sum \limits_{{{\text{j}} = 1}}^{\text{N}} \mathop \sum \limits_{{{\text{j}} \ne {\text{k}},{\text{k}} = 1}}^{\text{N}} {\text{p}}_{\text{ijk}}$$

The SPS for the alignment is given by

2 $${\text{SPS}} = \sum_{{{\text{i}} = 1}}^{{\text{M}}} {{\text{S}}_{{\text{i}}} } {\bigg/}\sum_{{{\text{i}} = 1}}^{{{\text{M}}_{{\text{r}}} }} {{\text{S}}_{{{\text{ri}}}} }$$

where ${\text{M}}_{\text{r}}$ is the number of columns in the reference alignment and ${\text{S}}_{\text{ri}}$ is the score ${\text{S}}_{\text{i}}$ for the *i*th column in the reference alignment.

### BAliBASE3 database

With the evolution of the sequence and structure databases resulting from high throughput technologies, the multiple alignments of large numbers of complex, multi-domain sequences have become a standard requirement. Sequence alignment benchmarks must not only evolve to accurately represent the requirements, but also to avoid over-fitting of the methods to a particular set of test cases. BAliBASE release 3.0 is designed to respond to these challenges (Thompson et al. 2005). The size of the alignments in the BAliBASE benchmark has been increased in release 3.0 to reflect the ever-growing sequence and 3D structure databases. Furthermore, because the reference sequences in the database are manual comparison, the results are more biological characteristics and they are common databases of test algorithm.

The BAliBASE 3.0 contains 218 reference alignments shown in Table 2, which are distributed into five reference sets. Reference set 1 is a set of equal-distant sequences, which are organized into two reference subsets, RV11 and RV12. RV11 contains sequences sharing >20 % identity and RV12 contains sequences sharing 20–40 % identity. Reference set 2 (RV20) contains families with >40 % identity and a significantly divergent orphan sequence that shares <20 % identity with the rest of the family members. Reference set 3 (RV30) contains families with >40 % identity that share <20 % identity between each two different sub-families. Reference set 4 (RV40) is a set of sequences with large N/C –terminal extensions. Reference set 5 (RV50) is a set of sequences with large internal insertions.

**Table 2:** BaliBASE 3.0 Statistics

	RV11	RV12	RV20	RV30	RV40	RV50	TOTAL
Number of alignment	38	45	41	30	48	16	218
Number of sequence	265	411	1896	1882	1317	483	6255

## Results

### Experiment setting

In the experiment, we use the database of BaliBASE 3.0 shown in Table 2.

To assess the performance of the formulas in this study, SPS (sum-of-pair score) is as objective function. The SPS is calculated such that the score increases with the number of sequences correctly aligned (Thompson et al. 1999). It is used to determine the extent to which the programs succeed in aligning some, if not all, of the sequences in an alignment. If the SPS is higher, the results of alignment are closed to reference alignment and even better than the reference alignment.

**Fig. 1:**
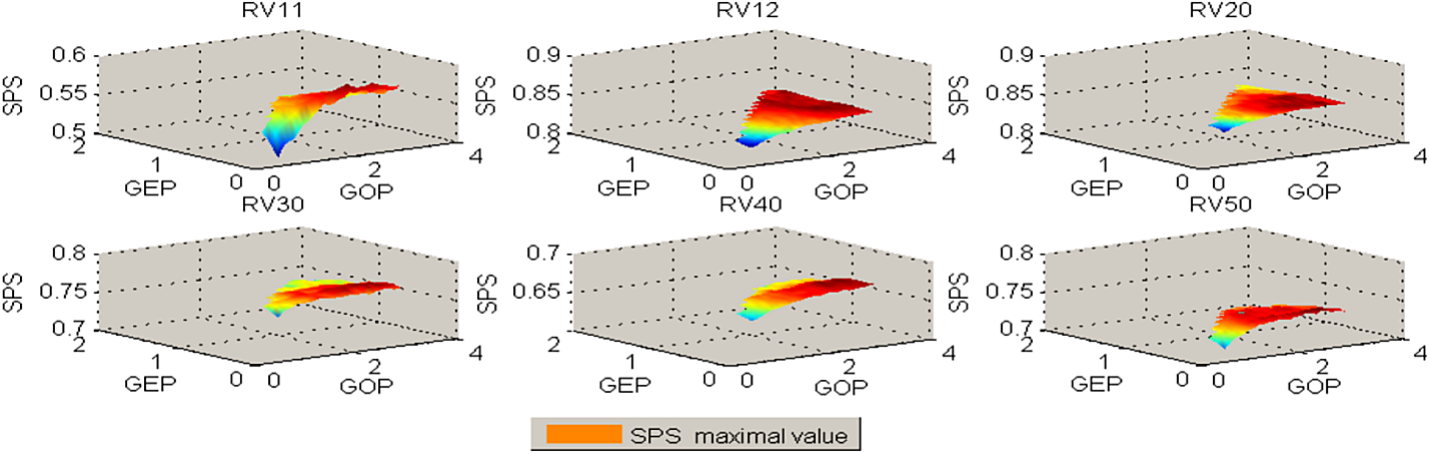
The value of SPS

To obtain the optimal parameters combination of MAFFT program, we used batch processing through Perl programming (ActivePerl 5.16.2 version) language on Windows7 OS: the step of GOP is 0.1, the step of GEP is 0.03, the SM is BLOSUM30/BLOSUM45/BLOSUM62/BLOSUM80/PAM100/PAM200 respectively. The batch processing script is following: 

For each alignment of BaliBASE 3.0, the number of alignment results is 692, because there are 692 kinds of parameters combination pattern. For each combination of the three parameters (SM, GOP, GEP), each alignment of reference can obtain SPS score. Figure 1 illustrates all the SPS results of six References. In each of these graphs, the SM is BLOSUM45/BLOSUM45/BLOSUM62/BLOSUM45/BLOSUM80/BLOSUM45 respectively. The SPS reaches the maximal value when the GOP, GEP and SM is certain value respectively.

### The determination of optimal substitution matrix parameters

The determination of the optimal matrix is as follows:

1. Compute SP scores of each substitution matrix according to the parameters setting. For each substitution matrix, GOP and GEP have 692 different combination modes, so the number of SP score is (692× the number of reference alignment). For example, RV11 has 38 reference alignments, so the number of SP scores is 38 × 692.
2. Compute the mean value of SP scores in each GOP/GEP combination mode, which is denoted by MEAN_SPS. For example, the number of SP scores of RV11 is 38 × 692, so the number of MEAN_SPS is 1 × 692.
3. Compute the maximum value of MEAN_SPS, which is denoted by MAX_MEAN_SPS. For example, the number of MEAN_SPS of RV11 is 1 × 692, so the number of MAX_MEAN_SPS is 1. The greater the MAX_MEAN_SPS value, the higher the alignment accuracy in the GOP/GEP combination mode.
4. The MAX_MEAN_SPS values with each substitution matrix and each data set are listed in Table 3.

**Table 3:** The MAX_MEAN_SPS value of different substitution matrix

Data set	RV11	RV12	RV20	RV30	RV40	RV50
BLOSUM30	0.5201	0.8369	0.8532	0.7683	0.6688	0.7427
BLOSUM45	*0.5912*	*0.8465*	0.8577	0.7727	*0.6818*	*0.7505*
BLOSUM62	0.5791	0.8380	*0.8594*	*0.7819*	0.6745	0.7466
BLOSUM80	0.5770	0.8396	0.8573	0.7737	0.6752	0.7468
PAM100	0.5453	0.8315	0.8535	0.7694	0.6686	0.7423
PAM200	0.5415	0.8309	0.8518	0.7728	0.6682	0.7348

Italic figures represent the best results
5. The maximum value of MAX_MEAN_SPS is corresponding to the optimal matrix. Bold figures represent the best results.

### The determination of optimal GOP/GEP parameters

The determination of the optimal GOP and GEP is as follows: the best MAX_MEAN_SPS values of each data set can be obtained, and they are corresponding to the certain GOP and GEP values (Table 3). As shown in Table 4, parameters obtained from our experiments are different from MAFFT default parameters.

**Table 4:** The optimal parameters for each data set

Data set	The optimal substitution matrix	The best MAX_MEAN_SPS value	The optimal GOP	The optimal of GEP
RV11	Blosum45	0.5912	2	0.12
RV12	Blosum45	0.8465	2.9	1.44
RV20	Blosum62	0.8594	2.3	0.63
RV30	Blosum62	0.7819	2.1	0.72
RV40	Blosum45	0.6818	2.8	0.39
RV50	Blosum45	0.7505	2.8	0.03

### The mean of SPS obtained from different algorithm

**Fig. 2:**
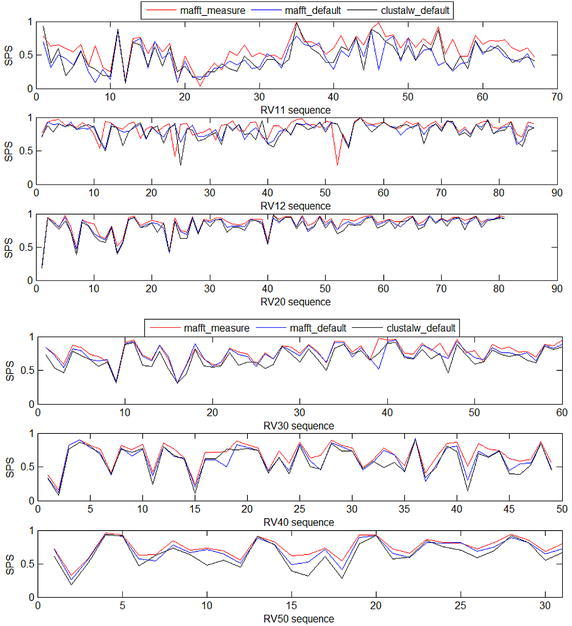
The SPS values of sequence sets in different algorithm

Table 5 shows the mean of SPS values from different algorithm. The SPS value obtained from MAFFT default parameters is higher than the CLUSTALW (CLUSTALW-2.1-WIN) default parameters. The SPS value of MAFFT measure parameters is higher than MAFFT default parameters. Figure 2 illustrates the SPS value of MAFFT measure parameters, MAFFT default parameters and CLUSTALW default parameters. For set of sequences, the SPS value is the best in MAFFT measure parameters.

**Table 5:** The mean of SPS value from different algorithms

	RV11	RV12	RV20	RV30	RV40	RV50
mafft-default	0.4582	0.8142	0.8301	0.737	0.6168	0.6971
clustalw-default	0.4758	0.7966	0.8077	0.6802	0.5917	0.6377
mafft_measure	*0.5912*	*0.8465*	*0.8594*	*0.7819*	*0.6818*	*0.7505*

Italic figures represent the best SPS value from MAFFT measure parameters

## Discussion

In this paper, we use MAFFT tool to improve MSA. In order to get better SPS results, we abandon the default parameters in the process of MSA, and seek to find the optimal parameter combination. Experimental results show that the MSA results highly depend on the substitution matrix and gap penalties. Applying MAFFT tool with optimal parameter combination, we find that the accuracy of MSA result is higher than MAFFT and ClustalW with default parameters. This study allows to optimize the multiple sequence alignment results and provides a new idea for multiple sequence alignment.

In the future work, firstly, we can use these proposed formulas and similar method to find optimal parameter combination of other MSA tools, such as CLUSTALW, MUSCLE and so on. Secondly, the article mainly discusses the optimal parameters combination of MAFFT program based on BAliBASE3.0 database. Because the reference sequences in the BAliBASE are manual comparison, the alignment is more biological characteristic, and it is one of the common databases of test algorithm. In the future, we will discuss the other benchmarks to find the optimal parameters of MAFFT. Maybe, the default parameters are the best results for MAFFT program on other benchmarks. However parameter of MAFFT program is improved, the research is not ended.
